# Fragility Fractures Unveil the Hidden Dragon: A Case of Osteitis Fibrosa Cystica and Secondary Hyperparathyroidism in End-Stage Renal Disease Post-Trauma

**DOI:** 10.7759/cureus.58208

**Published:** 2024-04-13

**Authors:** Alexander Konopnicki, Servando Cuellar, Sarah Navid, Metha R Chea, Moe Ameri, Melinda B Tanabe

**Affiliations:** 1 Internal Medicine, University of Texas Medical Branch, Galveston, USA; 2 Internal Medicine, University of Texas Medical Branch John Sealy School of Medicine, Galveston, USA; 3 Infectious Disease, University of Texas Medical Branch, Galveston, USA

**Keywords:** healthcare disparity, incarcerated population, end-stage renal disease, fragility fracture, hyperparathyroidism, osteitis fibrosa cystica, brown tumor

## Abstract

Secondary hyperparathyroidism is a prevalent complication of end-stage renal disease (ESRD), arising from chronic renal insufficiency leading to disturbed calcium metabolism. This disruption triggers hypersecretion of the parathyroid gland, characterizing the condition. Osteitis fibrosa cystica (OFC), a rare complication of untreated secondary hyperparathyroidism, results in benign resorptive bone lesions and the formation of cystic cavities within bones. Our case report describes a 46-year-old incarcerated Hispanic male with a 17-year history of end-stage renal disease and secondary hyperparathyroidism. The patient initially presented with a traumatic right elbow injury. Further diagnostic evaluation revealed an 8 cm destructive process involving the distal humerus, initially suspected as malignancy but confirmed as OFC through bone biopsy. Management involved orthopedic surgery performing an open reduction and internal fixation (ORIF) of the affected limb, with subsequent consideration for inpatient parathyroidectomy. Imaging studies, including magnetic resonance imaging (MRI) and computed tomography (CT) scans, elucidated a 6 × 5.5 cm soft tissue mass, further confirmed as a brown tumor. The case underscores the complexities of diagnosing OFC, often misinterpreted in radiologic studies, and highlights the multidisciplinary approach involving orthopedics, otolaryngology, and nephrology in managing this intricate scenario. The objective is to explore clinical manifestations and treatment challenges of OFC and secondary hyperparathyroidism triggered by trauma in end-stage renal disease, emphasizing the need for continued awareness and precise diagnostic strategies in resource-rich areas.

## Introduction

Secondary hyperparathyroidism is a common complication of end-stage renal disease (ESRD) [1].​ Its pathogenesis lies in chronic renal insufficiency, which manifests as decreased calcium, elevated phosphate, and impaired metabolism of 1,25-(OH)2D3 (calcitriol). These disruptions prompt hypersecretion of the parathyroid gland, characterizing the condition [2].​ Osteitis fibrosa cystica (OFC) emerges as a rare complication in patients with untreated secondary hyperparathyroidism in end-stage renal disease. OFC develops due to hyperparathyroidism-induced benign resorptive bone lesions, resulting in the formation of cystic cavities within bones [3].​ These cavities are filled with fibrinous tissue exhibiting a distinctive brown hue, classically characterized as a brown tumor [4].​ While historically used interchangeably, brown tumor and OFC refer to distinct aspects of the disease process. OFC refers to the pathophysiologic changes occurring, while brown tumor describes the histopathological appearance of fibrinous tissue. Renal osteodystrophy is a complication of chronic kidney disease marked by bone abnormalities due to imbalances in calcium, phosphate, and vitamin D, leading to bone pain, fractures, and deformities [5].​ Treatment focuses on dietary and pharmacological management of these mineral levels to mitigate bone disease and related complications. Renal osteodystrophy is now less common due to precise serum calcium monitoring, allowing early detection in resource-rich areas. The prevalence of bone lesions has decreased from 80% to 15% [6].^​^ Radiologic evidence of OFC is rare and often leads to misdiagnoses on computed tomography (CT) imaging, commonly confused with conditions such as metastatic carcinoma, bone cysts, osteosarcoma, and giant cell tumors [7].​ Here, we report a case of a male with a history of end-stage renal disease presenting with a traumatic fracture. Upon investigation, it was revealed to be a mere shadow concealing a brown tumor, leading to the subsequent diagnosis of osteitis fibrosa cystica.

## Case presentation

A 46-year-old incarcerated Hispanic male was admitted for evaluation of a traumatic right elbow fracture. The patient's right arm was injured when an individual ran into his right arm outstretched between the gaps of his prison cell's bars. The patient reported mild numbness and tingling associated with his right arm pain. He denied any weakness of the right upper extremity and maintained an adequate range of motion of his hand and wrist. Additionally, he endorsed diffuse bone pain over the past seven years and reported a similar event when he fractured his left arm several years prior. On physical examination of the right upper extremity, there was a firm mass on the lateral distal humerus with moderate tenderness to palpation. Sensation was intact to light touch with 5/5 strength.

His past medical history was significant for end-stage renal disease (ESRD) due to long-standing uncontrolled hypertension from a very young age on hemodialysis for the past 17 years, secondary hyperparathyroidism, and a benign right thyroid mass status post a partial thyroidectomy of the benign right thyroid mass. Social history was largely non-contributory but included a remote 2.5-pack-year smoking history. He had no pertinent family history.

The patient's end-stage renal disease and secondary hyperparathyroidism were managed as an outpatient by nephrology. His home medications included calcitriol 2 mcg daily, cinacalcet 60 mg in the morning and 90 mg in the evening, and Phoslo 667 mg twice a day. He was on a Monday, Wednesday, and Friday hemodialysis schedule, which was continued while inpatient.

**Figure 1:**
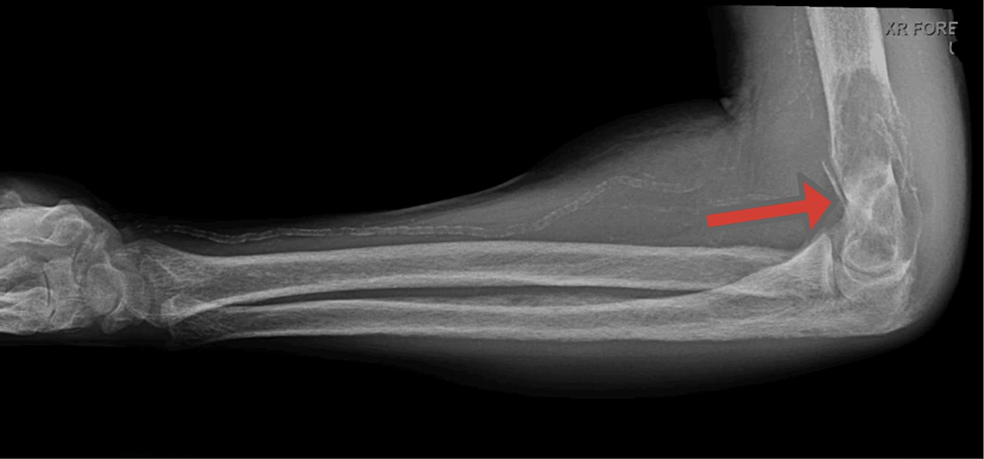
Two-view right forearm X-ray: 8 cm destructive process in the distal meta-diaphysis of the humerus with pathologic fracture, cortical-based focus on the proximal ulna, olecranon enthesopathy, and advanced vascular calcification (arrow)

**Figure 2:**
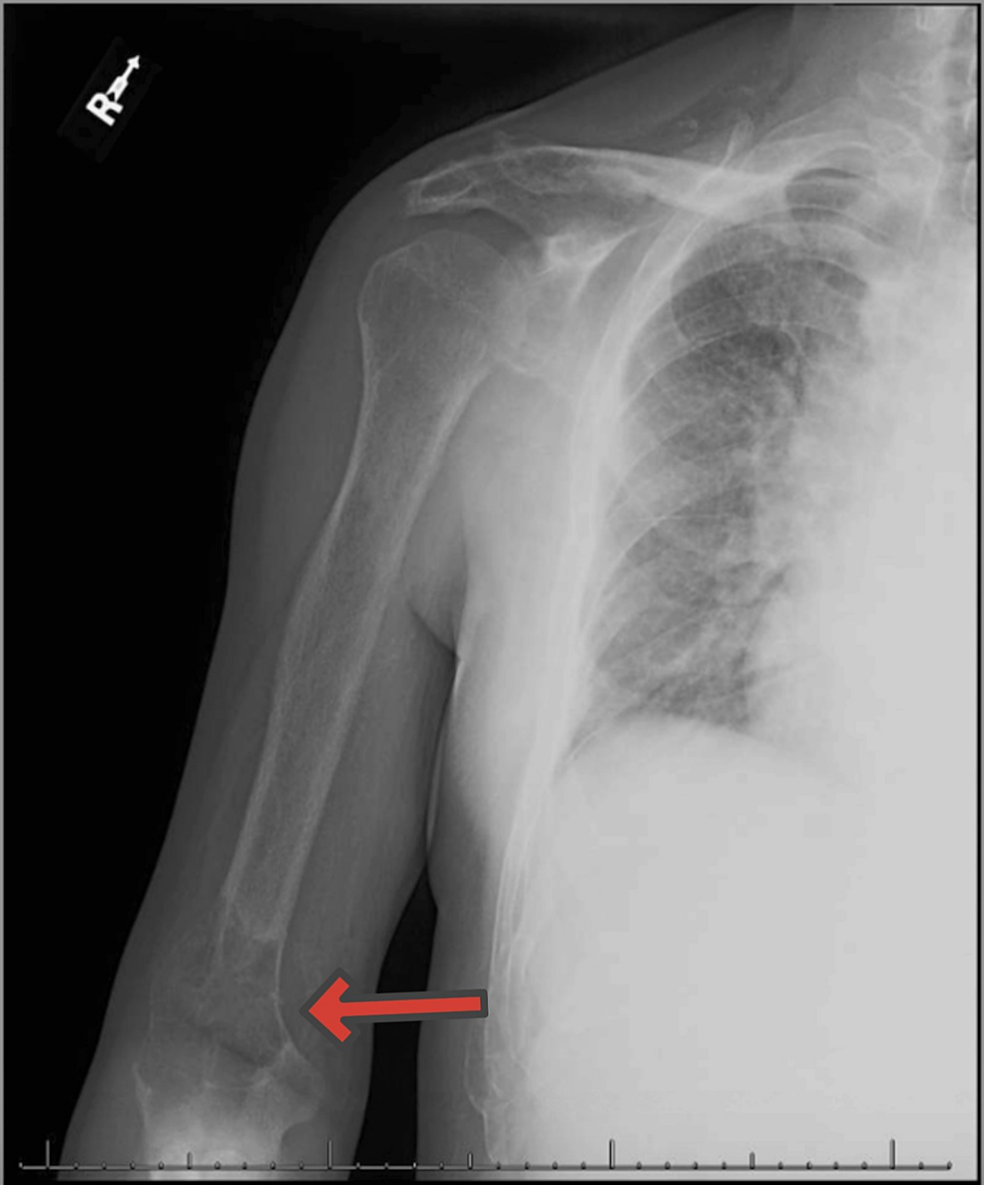
Two-view right humerus X-ray: 8-cm destructive process in the distal meta-diaphysis of the humerus with pathologic fracture, cortical-based focus on the proximal ulna, olecranon enthesopathy, and advanced vascular calcification (arrow)

On admission, his laboratory results were notable for a parathyroid hormone (PTH) of 2182.6 mmol/L, and his previous PTH levels were spare, with a calcium of 6.8 mg/dL. A two-view X-ray of the right humerus (Figure 1) and a two-view X-ray of the right forearm (Figure 2) showed an 8 cm destructive process involving the distal meta-diaphysis of the humerus with involvement of the entire diameter and a displaced pathologic fracture.

**Figure 3:**
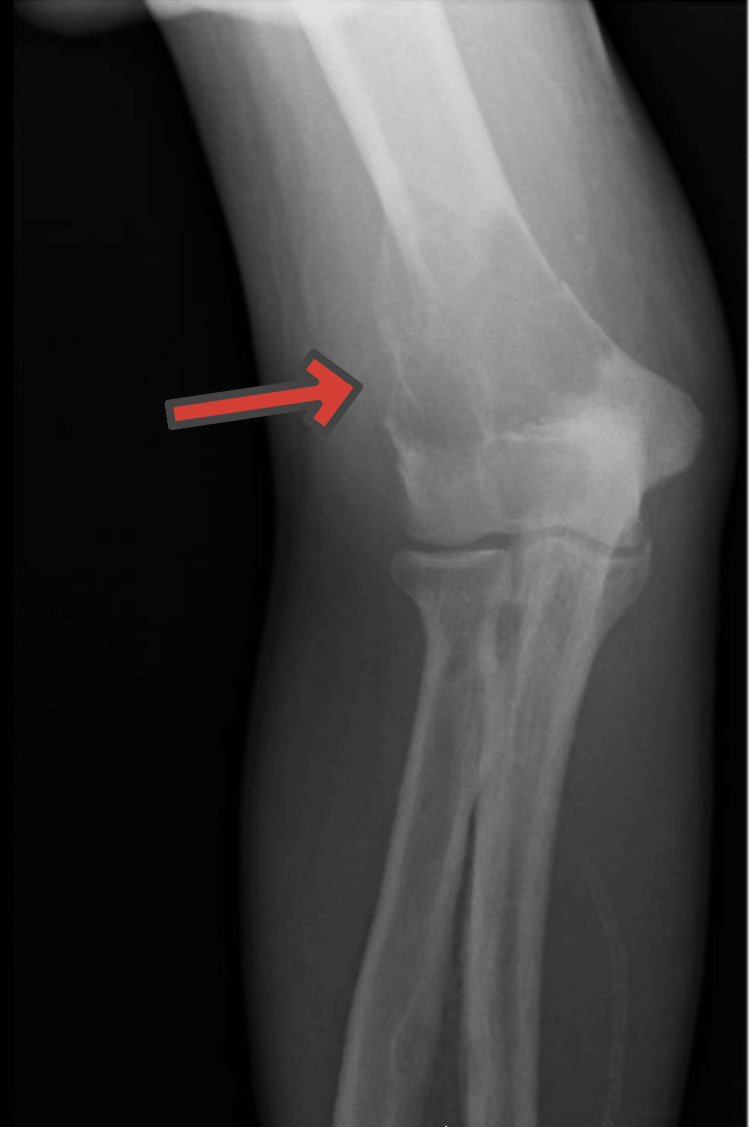
Two-view right elbow X-ray: 5 cm destructive process of the distal meta-diaphysis of the humerus with involvement of the entire diameter and mildly displaced pathologic fracture, cortical-based focus with peripheral sclerosis at the proximal diaphysis of the ulna, olecranon enthesopathy, soft tissue edema, and advanced vascular complication (arrow)

A two-view X-ray of the right elbow (Figure 3) showed a 5 cm destructive process at the distal meta-diaphysis of the humerus with pathologic fracture, concerning for malignancy or metastatic disease.

**Figure 4:**
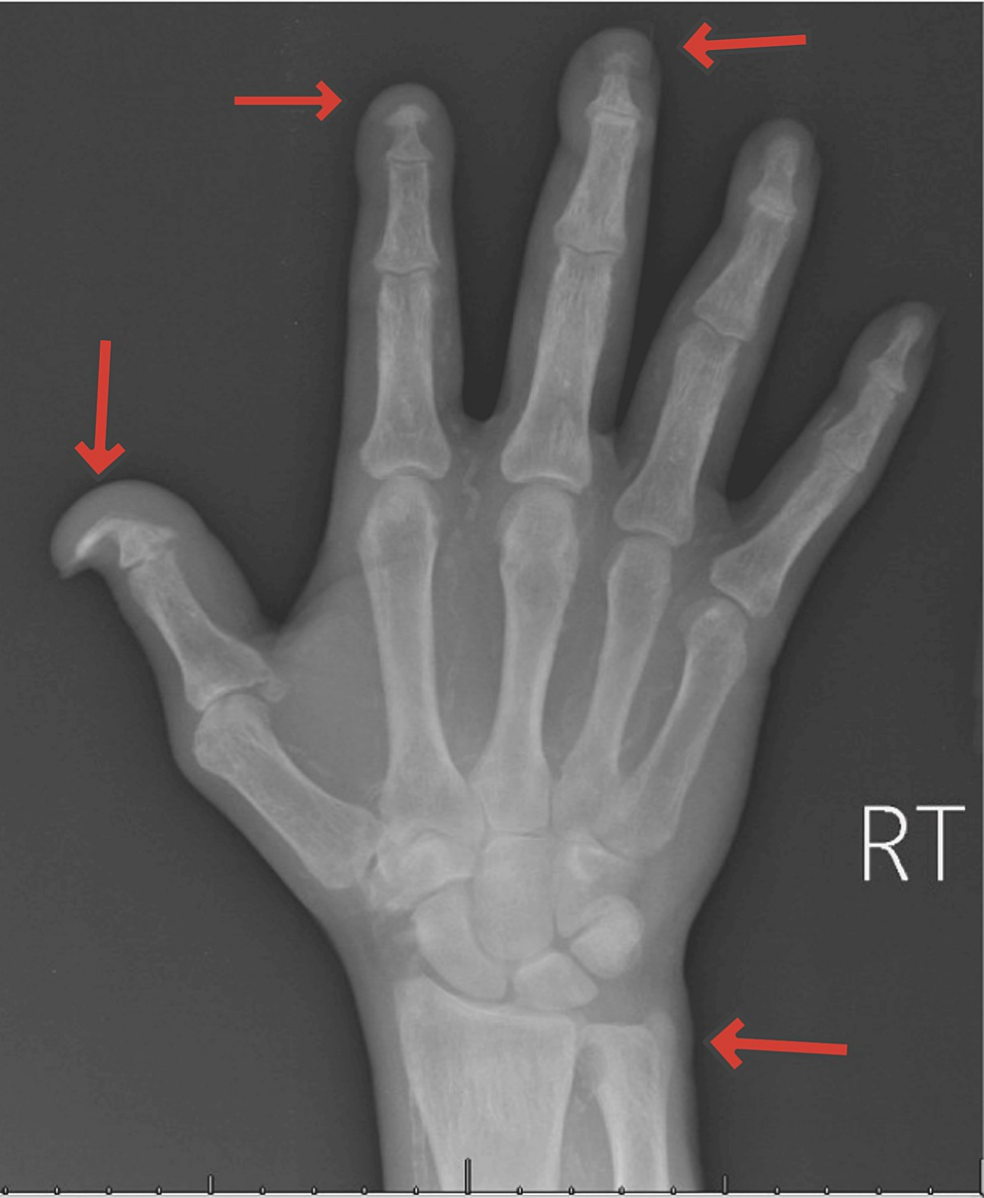
Three-view right hand X-ray: resumption versus osteolytic process at the distal ulna at the distal radioulnar joint, as well as chronic resumption and/or post-traumatic changes of the first, second, and third distal phalanges (arrows)

A three-view X-ray of the right hand (Figure 4) showed resumption versus an osteolytic process at the distal ulna at the distal radioulnar joint, which could represent inflammatory arthropathy or lytic metastasis. It also showed chronic resumption and/or post-traumatic changes of the first, second, and third distal phalanges.

**Figure 5:**
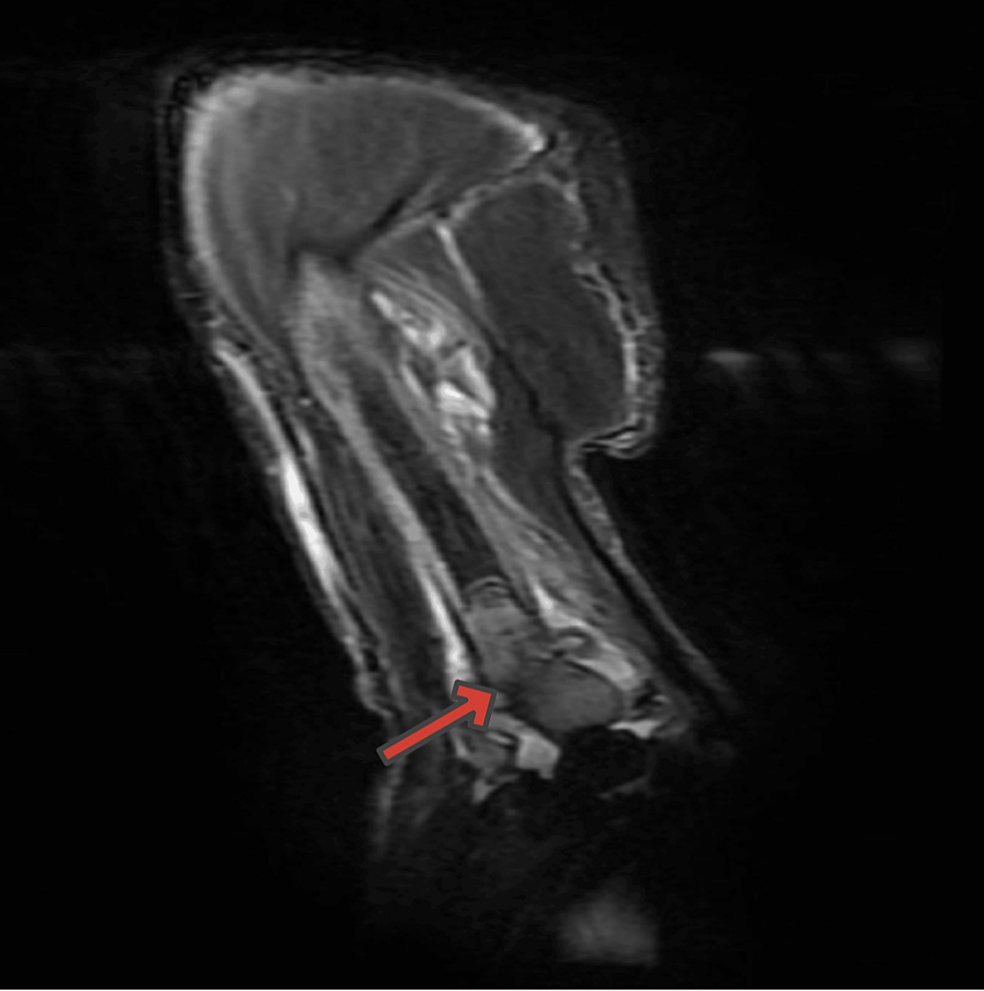
Right humerus MRI: complete destruction of the distal 7 cm of the right humerus manifested by enhancing low T1 signal intensity with cortical destruction and associated soft tissue component (6 × 5.5 cm) (arrow) MRI: magnetic resonance imaging

Magnetic resonance imaging (MRI) of the right humerus (Figure 5) showed complete destruction of the distal 7 cm of the right humerus, compatible with malignancy and a large elbow joint effusion.

**Figure 6:**
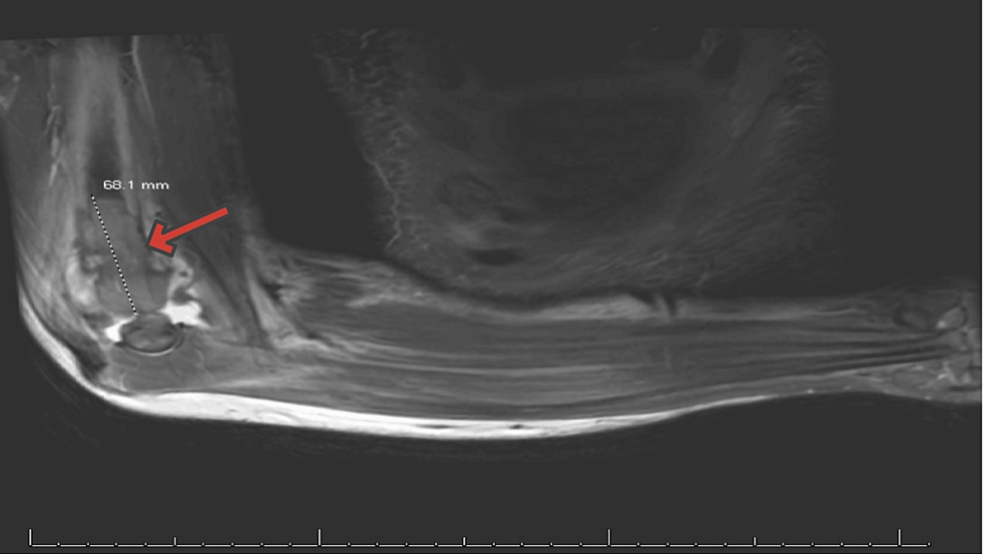
Right forearm MRI: expansile hypointense T1/T2 signal lesion at the distal humeral meta-diaphysis with avid enhancement measuring 6.8 × 5.3 × 5 cm (arrow), moderate elbow effusion is present, musculatures of the right forearm demonstrate preservation of signal intensity without fatty infiltration or denervation atrophy, and visualized joint spaces are preserved MRI: magnetic resonance imaging

MRI of the right forearm (Figure 6) showed an expansile hypointense T1/T2 signal lesion at the distal humeral meta-diaphysis with avid enhancement, measuring 6.8 × 5.3 × 5 cm. 

The differential diagnosis based on the initial radiology readings included a giant cell tumor, chondrosarcoma, or a metastatic process. Another consideration high on the differential diagnosis based on our patient's past medical history was osteitis fibrosa cystica (OFC). A bone biopsy was obtained, which did not show any sign of malignancy and confirmed the lesion as OFC from uncontrolled secondary hyperparathyroidism. Management included orthopedic surgery, performing an open reduction and internal fixation (ORIF). Otolaryngology was consulted for evaluation for possible inpatient parathyroidectomy. The decision was made for the patient to first recover from his ORIF before pursuing further inpatient workup of his secondary hyperparathyroidism, which would have included serum calcium, phosphate, parathyroid hormone levels, and vitamin D status, and targeted imaging studies such as neck ultrasound, sestamibi scan, and bone density scans to assess parathyroid gland activity and bone disease and possible parathyroidectomy. The patient was discharged back to his unit with referrals placed to otolaryngology, orthopedic surgery, and nephrology.

## Discussion

Our case report delves into the intricate interplay of secondary hyperparathyroidism and osteitis fibrosa cystica (OFC) in an individual with end-stage renal disease (ESRD) following trauma. Secondary hyperparathyroidism, a common consequence of ESRD, arises from the disruption of calcium homeostasis due to chronic renal insufficiency. This disruption prompts hypersecretion of the parathyroid gland, leading to increased levels of parathyroid hormone (PTH) and subsequent bone resorption. OFC, a rare complication of untreated secondary hyperparathyroidism, manifests as benign resorptive bone lesions, often resulting in cystic cavities within bones. Our patient, an incarcerated Hispanic male with a prolonged history of ESRD, presented with a traumatic right elbow injury, unveiling a complex scenario of fragility fractures and OFC.

The case emphasizes the diagnostic challenges associated with OFC, which often presents asymptomatically in the early stages, leading to delayed diagnoses. Radiographically, well-defined lytic lesions with cortical thinning are characteristic, underscoring the importance of thorough imaging and pathological assessment. The management of OFC involves a multidisciplinary approach, including orthopedic surgery for interventions such as open reduction and internal fixation (ORIF), otolaryngology for potential parathyroidectomy, and nephrology for the management of secondary hyperparathyroidism.

Continuous monitoring of biochemical markers such as PTH, vitamin D, phosphate, and total calcium is essential for effective disease management. Our patient's clinical course, from the initial suspicion of malignancy to the confirmation of OFC through bone biopsy, highlights the critical role of accurate diagnosis in guiding appropriate interventions. Furthermore, the case underscores the significance of heightened awareness among healthcare professionals about the varied presentations of OFC in imaging studies, preventing misdiagnoses commonly associated with conditions such as metastatic carcinoma, bone cysts, osteosarcoma, and giant cell tumors.

Incarceration adds a layer of complexity to the management of patients with ESRD, contributing to weakened bones and increased susceptibility to fractures. The discussion of treatment options, including parathyroid resection, becomes pivotal in optimizing the patient's long-term outcomes. This case report serves as a valuable contribution to the existing medical literature, shedding light on the diagnostic approach and therapeutic considerations in addressing OFC and secondary hyperparathyroidism in the context of trauma and ESRD in a unique patient population.

## Conclusions

In summary, this case study sheds light on the demanding aspects of diagnosing and treating osteitis fibrosa cystica (OFC) in the vulnerable and at-risk population of incarcerated individuals. In this population, usually manageable conditions such as end-stage renal disease (ESRD) can lead to seldomly seen complications such as fragility fractures from long-term hyperparathyroidism causing bone weakening. This case emphasizes the need for detailed patient history and a contextual understanding of comorbidities to diagnose rare bone disorders such as OFC. The case also illustrates the importance of a multidisciplinary team encompassing several specialties working together to manage each component of the complications that stemmed from ESRD. It offers insight into strategies for handling challenging cases involving chronic renal failure, secondary hyperparathyroidism, and bone health. There is a need for further research and education in this specialized field of medicine.
